# Epidemiological study in a small rural area of Veneto (Italian region) during Sars-Cov-2 Pandemia

**DOI:** 10.1038/s41598-021-02654-9

**Published:** 2021-12-01

**Authors:** Marco Bassanello, Luciano Pasini, Marco Senzolo, Andrea Gambaro, Marco Roman, Ugo Coli, Maurizio D’Aquino

**Affiliations:** 1 Covid Manager and Head of Accident and Emergency, Monastier di Treviso Hospital, Veneto, Italy; 2 Head of Laboratory and Microbiology, Monastier di Treviso Hospital, Veneto, Italy; 3 Multivisceral Transplant Unit, Department of Surgery, Oncology and Gastroenterology, Padua University Hospital, Padua, Italy; 4 Department of Environmental Sciences, Informatics and Statistics (DAIS), Ca’ Foscari University of Venice, Venice Mestre, Italy; 5 Health Director Monastier di Treviso Hospital, Veneto, Italy; 6 Head of Medicine Unit and Medical Department, Monastier di Treviso Hospital, Veneto, Italy

**Keywords:** Disease prevention, Public health, Epidemiology, Population screening

## Abstract

The emergence of severe acute respiratory syndrome type 2 coronavirus (SARS-CoV-2) and its complications have demonstrated the devastating impact of a new infectious pathogen. The organisational change promulgated by the isolation of affected communities is of extreme importance to achieve effective containment of the contagion and good patient care. The epidemiological study of the population of a small rural community in the North East of Italy revealed how much the virus had circulated during Spring, 2020, and how contagion has evolved after a prolonged lockdown. In the 1st phase, NAAT (Nucleic Acid Amplification Testing) was performed in cases with more or less severe symptoms and a study was performed to trace the infection of family members. Only 0.2% of the population tested positive on NAAT, via nasopharyngeal swab during this 1st phase. In the 2nd phase a random sample of the general population were tested for circulating anti-Sars-Cov-2 immunoglobulins. This showed that approximately 97.9% of the population were negative, while 2.1% (with positive IgG at a distance) of the population had contracted the virus in a mildly symptomatic or asymptomatic form. The main symptom in subjects who developed immunity was fever. Antibodies were found in subjects with forced coexistence with quarantined or infected subjects. The mutual spatial distance by categories has shown higher relative prevalence of IgG positive and IgM negative cases in close proximity but also far from the infected, with respect to an intermediate distance. This suggests that subjects living in thinly populated areas could come in contact with the virus more likely due to intentional/relational proximity, while those living nearby could also be infected through random proximity.

## Introduction

The 2019 Severe Acute Respiratory Syndrome (SARS) is caused by Coronavirus type 2 (CoV-2)^1^. The first cases were documented in China (in the Wuhan province) in late 2019 and early 2020^2^. The clinical presentation of CoV-2 infection, named COVID 19, is extremely varied, ranging from completely asymptomatic and / or minor respiratory and gastro-intestinal symptoms to flu-like syndrome, high fever, coughing, shortness of breath, muscle pain, tiredness^3,4^ and diarrhea^5^. SARS associated with high risk of death needing admission into intensive care units occurs only in a minority of cases^6,7^. An infected person may develop symptoms after an incubation period that can vary between 2 to 14 days (though up to 29 days has been reported). During this period, they can still be contagious^6,7^. Vaccines are currently available and widespreadly used in Europe however, limiting the transmission of the virus and its spread is still important given the possible recurrence of COVID19 waves due to the diminished level of protection over time. Symptomatic or infected subjects must remain in quarantine and contact a doctor immediately to receive the appropriate indications in case of fever persisting above 37.5 with a dry cough (9–3). In March 2020, the World Health Organisation (WHO) proclaimed Europe as the centre of the pandemic. Complete isolation was necessary because of the quick dispersion of the virus and our lack of knowledge about it. But there is now evidence that complete isolations of entire communities may be not necessary to control viral transmission and specific measures can be taken instead^8–10^.

As of November 2021, there are nearly 250 million confirmed cases globally since the pandemic started, with peaks in the Americas, Asia, and Europe. With over 5 million deaths reported, the risk of further escalation of Covid-19 is moderate to high despite Vaccinations and current Sars-Cov-2 knowledge (Source OME: Health Emergency Dashboard). Recommendations of the WHO on pandemic containment include the widespread use of diagnostic tests, quarantine of cases, contact traceability and social distancing. These are the basic principles of public health and infectious disease control (The Lancet editorial 2020). Because SARS-CoV-2 is a new pathogen, the characteristics of its transmission and diffusion are not yet well known. These include the change of R0 over time and in populations, the heterogeneity of the attack, the percentages of contact between the demographic groups, the interpersonal variation in the communicability and severity of the disease. All are essential to completely understand the spread of SARS-CoV-2 infection within different communities. Differences in population density, cultural and organisational behavior, population age, co-morbidity and contact rates between groups all influence the transmission dynamics within communities. Accordingly, the assumption of a uniform R0 among populations is unrealistic. Furthermore, variation in transmissibility between individuals can play an important role in the spread of SARS-CoV-2. It is unclear to what extent humans are able to generate SARS-CoV-2 immunity.

Reinfections are rare events and patients who have recovered from COVID-19 have a lower risk of reinfection. Natural immunity to SARS-CoV-2 appears to confer a protective effect for at least a year, which is similar to the protection reported in recent vaccine studies. However, the observation ended before SARS-CoV-2 variants began to spread, and it is less clear how much natural immunity to the wild-type virus will protect against variants^11^. Specific antibodies for serum neutralization (NAbs) for SARS-CoV-2 were detected in considerable, although variable, titers in most individuals^12^, indicating that NAb production against SARS-CoV -2 is relatively common. While these results are promising, other important questions to consider are whether NAb securities will decrease over time and how long acquired immunity will last. Previous studies in confirmed SARS patients have shown that NAb responses against SARS-CoV persisted from several months up to 2 years^13^. Mass serological testing is therefore needed to estimate how many individuals have been infected, how many are immune and how far we are from reaching the immunity threshold^14^. The WHO currently recommends that COVID-19 diagnosis be performed by laboratories using SARS-CoV-2 virus-targeted molecular tests. Serological tests are very useful to define the attack rate and immunity in communities which remain a top priority for keeping the pandemic under control^15^. Rapid antigen tests have now the advantage of rapidity and low costs with high sensitivity and specificity^16^. Some studies also suggest better accuracy of the antibody tests if performed on processed venous blood compared to capillary blood^17^. Serological methods will play an important role in the epidemiology of COVID-19 and in determining the immune status of asymptomatic patients, and serology is helpful in combination with nucleic acid amplification tests for the most appropriate diagnosis of COVID-19^18^. The main tests used for SARS-CoV-2 are performed on nasopharyngeal and oropharyngeal swabs for categories of subjects with symptoms and those potentially exposed^19^. Nearly all immunocompetent persons develop an immune response following SARS-CoV-2 infection, including B and T cell-mediated immunity due to antiviral humoral and cellular immune responses, respectively. This includes antibodies directed against S and N proteins. Antibodies – including IgM, IgG, and IgA can be detected within 1–3 weeks after infection. IgM and IgG antibodies can arise nearly simultaneously; however, IgM antibodies decay more rapidly than IgG. The observed persistence of antibodies can vary by assay, and some studies have found that approximately 5–10% do not develop detectable IgG antibodies following infection^20^.

In the city of Wuhan the prevalence of IgG was 89.8% in COVID-19 patients, 4.0% in healthcare professionals, 4.6% in general workers and 1.0% in other patients. The prevalence of IgG increases significantly with age amongst healthcare professionals and family doctors. The prevalence of IgM antibodies to SARS-CoV-2 was 31.4% in subjects with COVID-19, 1.5% in health workers, 1.3% amongst general workers and 0.2% in other patients^21^.

In Veneto, at the end of the lockdown (from February 22nd to March 9th), there were 744 cases with a daily increase of 70 subjects per day; at the end of phase 1 (from March 10th to May 3rd) there were 18.318 cases and at the end of phase 2 (from May 4th to July 15th) there were 19.220 positive cases^22^. From 25 May to 15 July, a seroprevalence survey on SARS-CoV-2 was conducted in Italy by the national Statistics Office (Istat) and the Ministry of Health. The Red Cross undertook the operational work. The data show that 1,482,000 people of all encountered the virus, i.e. 2.5% of the population (Italian Government sources).

In order to study the epidemiology of COVID 19 in the population of a small rural municipality in the Veneto Region, the following was undertaken:

1. Analysis of symptomatic positive swab subjects during phase 1 of the pandemic.
2. Analysis of IgM and IgG during phase 2 from COVID-19 in a population of about 1000 subjects from a small town during the SARS—Cov—2 pandemic in Veneto (Italy). This analysis allows an extrapolated, indirect estimate of the subjects affected by the pandemic.
3. Association between acquired immunity and symptoms, previous quarantine or contacts from previous contagion.
4. Association between acquired immunity subjects and infected subjects.
5. Epidemiological trend within the family and social context.
6. Geospatial distribution patterns of the infection in a small municipality, namely Monastier di Treviso.

## Patients and methods

### Patients

During the Coronavirus Pandemic of 2020 in Italy, one can distinguish three different periods:

1. The lockdown phase from February 22nd to March 9th when the swab was made only on full-blown SARS, i.e. less than 0.5% of the infected, with no serological testing for antibodies;
2. Phase 1 from March 10th to May 3rd, when nasopharingeal swabs were started even in asymptomatic subjects and serological tests began to detect a poor immune response to the virus;
3. Phase 2 from May 4th to July 15th when all suspected COVID 19 individuals began to be swabbed; to monitor all health personnel and guests of nursing homes and hospitals or other health structures; and, especially, young people, even asymptomatic ones. During this phase there was a gradual release of personal and social constrains.

The recruitment for this investigation started on May 25th 2020.

The study involved one subject per family within a population of about 4400 people in the town of Monastier di Treviso (Veneto Region, Italy). This has approximately 1750 families, with an average of 2.49 subjects per family (source: Municipality of Monastier di Treviso). A total of 922 people was recruited (about a quarter of the area’s population). Recruitment was random and families remained free to participate; generally, the ones most at risk and the ones who had certainly been infected participated. The study was completed within 10 days. The epidemiological situation in Veneto at the end of phase 2 was characterised by Rt of 0.53 (among the lowest in Italy, according to Istituto Superiore Sanità). Patients were enrolled after informed consent was provided.

### Study protocol

Peripheral venous blood samples were collected. Specific qualitative determination of IgG and IgM antibodies directed against the 'new Coronavirus' plasma levels were measured by immunochromatographic method and a two-phase immuno-enzyme sandwich method with final fluorescence detection (ELFA).

Medical history information was collected at the time of sampling, namely: 1. presence of symptoms (none, fever, coughing, general malaise, diarrhea, flu symptoms, sore throat, nasal discharge, altered taste or smell)—even in the previous weeks/months; 2. relationship in the family (single, son, parent, grandfather, uncle, grandson, cousin, husband, wife); 3. previous quarantine or buffer due to risk factors; 4. contact with infected subjects through social and / or work activities.

All methods were carried out in accordance with relevant guidelines and regulations, respecting the Privacy of patients (The ethics committee of the Giovanni XXIII Hospital approved this study protocol # 12/2020 of 10 April, 2020).

### Statistical and geospatial analysis

Categorical data are described by frequency and percentage, continuous data by mean and standard error of the mean. Statistical analysis was performed using the Student *t* test for paired data. Simple regression was used for correlation analysis. Groups were compared using the unpaired Student *t-*test, analysis of variance for repeated measures, or the Fisher exact test, as appropriate. Statistical significance was set at *p* < 0.05.

Geospatial analysis was performed using QGis v3.14. The sampling density per km2 was calculated using Quartic (biweight) kernel estimator, which reliably mirrors population density. The same approach was used to calculate the spatial densities for individual variables including gender, age groups (< 30; 30–70; > 70), most significant symptoms, previous quarantine and contact cases, and IgG positive plus IgM negative cases. All variables densities were represented, both directly and normalised for the sampling density to overcome the masking of effect of the latter being strongly inhomogeneous (i.e. resulting in the percentage of specific cases relative to tested subjects per km2). The centroids of Voronoi polygons were calculated from the dataset, then interpolated using the cubic splines algorithm for visualization of the maps. Nearest neighbour analysis was performed to assess the degree of spatial association between location of immunized subjects and phase 1 infected patients, as follows: The distance matrix was calculated amongst each subject in the study group and the nearest previously infected patient. All study subjects were then grouped into five equally represented classes (by numerosity) of distance; then the prevalence of IgG positive plus IgM negative cases, and the mean age (and corresponding confidence intervals, Wilson 95%) were calculated for each class.

## Results

### Epidemiology of coronavirus infection during phase 1

**Figure 1:**
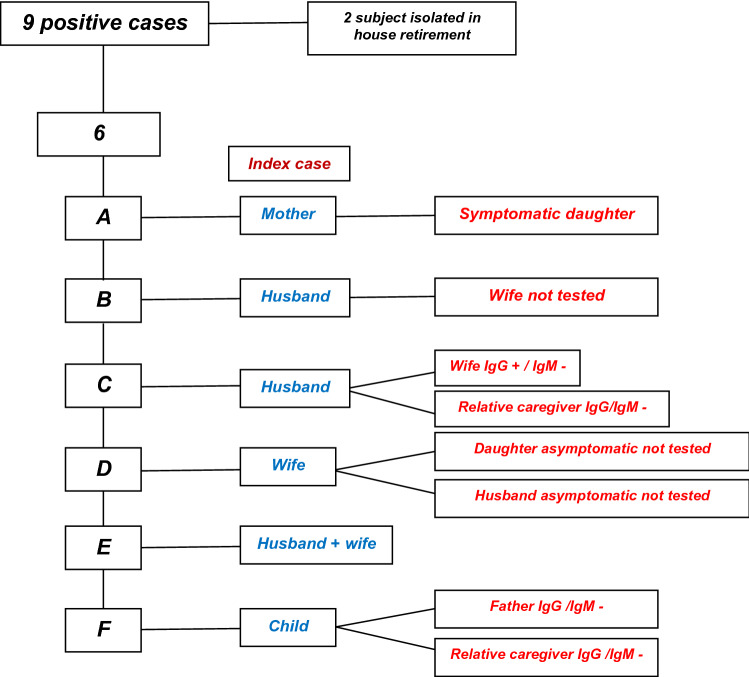
Family relationships diagram.

During the pandemic phase of COVID-19 in the Municipality of Monastier di Treviso, 9 positive cases were recorded on nasopharyngeal swab (as seen in the Family Relationships Diagram – Fig. 1). Of these, 3 were female and 6 were male. The average age was 58. Two of these subjects were housed in a retirement home: 1 positive patient was placed in quarantine inside the structure without ever coming into contact with family members or other guests of the home, and treated until negative. The other one arrived already swab negative. The 6 families of the positive subjects with COVID-19 and positive at the NAAT via swab were named A to F. In family A, the infected subject was the mother. One of the three daughters, who remained in contact and in quarantine with the infected mother, and who presented with a fever during that period, was positive for IgG antibodies and negative for IgM antibodies. In family B, the infected subject was the husband. The wife, the only person belonging to the same family unit, did not take the test. In family C, the husband was infected. He was hospitalised for respiratory complications by COVID-19. Meanwhile his wife, who remained in contact and in quarantine with her infected husband, but who was asymptomatic, was positive for IgG antibodies and negative for IgM antibodies. A relative, also asymptomatic, who cared for the family, was negative for both IgG and IgM antibodies. In family D, the infected subject was the wife. Her husband and daughter did not take the test. In family E, both spouses tested positive on nasopharyngeal swab, but did not take the test. In family F, the infected subject was one of the children, who remained in quarantine away from the rest of the family until complete recovery. The father who never had symptoms and saw his son after his quarantine, was found to be negative for both IgG and IgM antibodies. A relative who was always asymptomatic and who cared for the infected subject with protective equipment was also negative for both IgG and IgM antibodies. All infected subjects were subsequently negative on swabbing.

No subjects who tested positive on rhino-pharyngeal swab in the previous weeks presented for a second swab during serological screening in Phase 2.

### Prevalence of antibodies during phase 2

Out of a population of 951 enrollable subjects, 922 attended to undergo the test. At the time of the test, patients’ consent was obtained and clinical history data collected. These included: relationship in the family (single, son/daughter, parent, grandfather/grandmother, uncle/aunt, grandson, husband/wife/cohabiting partner); presence of symptoms, even in previous months (none, fever, illness, diarrhea, flu symptoms, sore throat, nasal discharge, alteration of taste or smell, cough); previous quarantine; previous contact with infected subjects. This information was not collected due to lack of consent or other issues for 271 subjects (29.4%). It was collected for 652 subjects (70.6%).

The characteristics of the study group and the outcome are reported in Table 1.

**Table 1:** Characteristics of the study group.

	All patients (n = 922)
Age (yr,mean ± SE )	55,9 ± 16,4
Gender (male/female)	452 (49%)/470 (51%)
*Relationship*	
Single	143 (15,5%)
Son/daughter	41 (4,4%)
Parent	225 (24,4%)
Grandfather/grandmather	10 (1,1%)
Uncle/aunt	1 (0,1%)
Grandson	2 (0,2%)
Husband	132 (14,3%)
Wife	103 (11,2%)
Not detected	265 (28,7%)
*Symptoms*	
None	522 (56,6%)
Fever	40 (4,3%)
Illness	3 (0,3%)
Diarrhea	3 (0,3%)
Flu symptoms	44 (4,8%)
Sore throat	18 (2,0%)
Nasal discharge	9 (1%)
Alteration of taste or smell	1 (0,1%)
Cough	18 (2,0%)
Not detected	264 (28,6%)
Quarantine	14 (1,5%)
Contact with infected subjects	12 (1,3%)
IgG positive	19 (2,1%)

**Figure 2:**
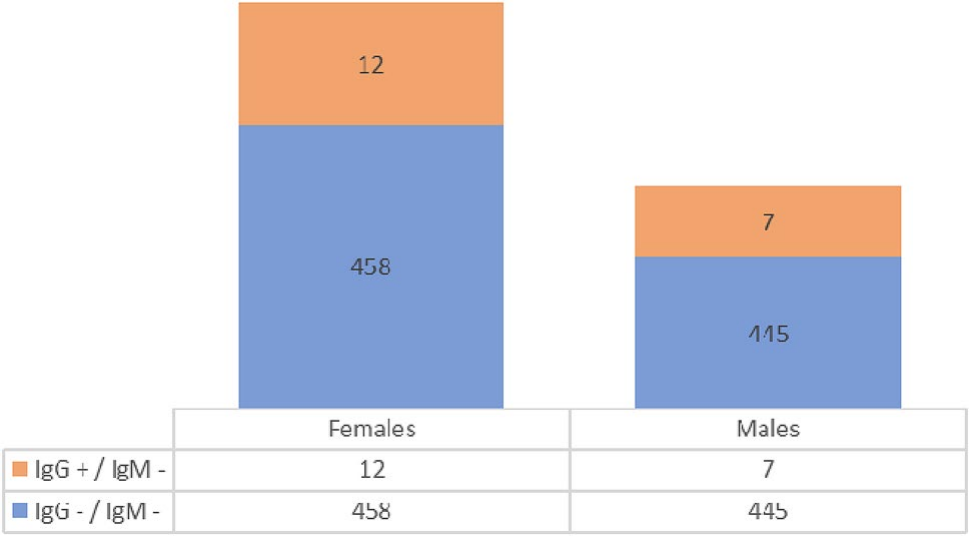
IgG and IgM antibodies and sex distribution.

The outcomes of IgG and IgM antibodies determination are reported in Table 1 and presented in Fig. 2. In 19 subjects (2.1%), the search for IgG antibodies was positive and the search for IgM antibodies was negative. In 903 subjects (97.9%) the search for IgG and IgM antibodies was negative. In no case was the IgM search positive.

**Figure 3:**
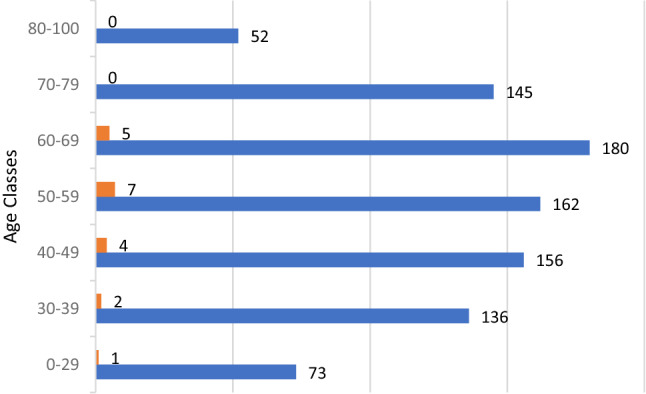
IgG and IgM antibodies and age distribution.

Analysis of the features of individuals with positive IgG reveals that the average age is 52 years, that under 30 s are only 2 and that those between 30–69 years number 17. There were no over 70 s. Females predominate over males (12/7). It is interesting to note that only fever (especially when associated with lack of taste or smell), quarantined subjects, the contact subjects of certain COVID-19, have a significant correlation in the seeric positivity to IgG. The distribution of patients by age is shown in Fig. 3.

**Figure 4:**
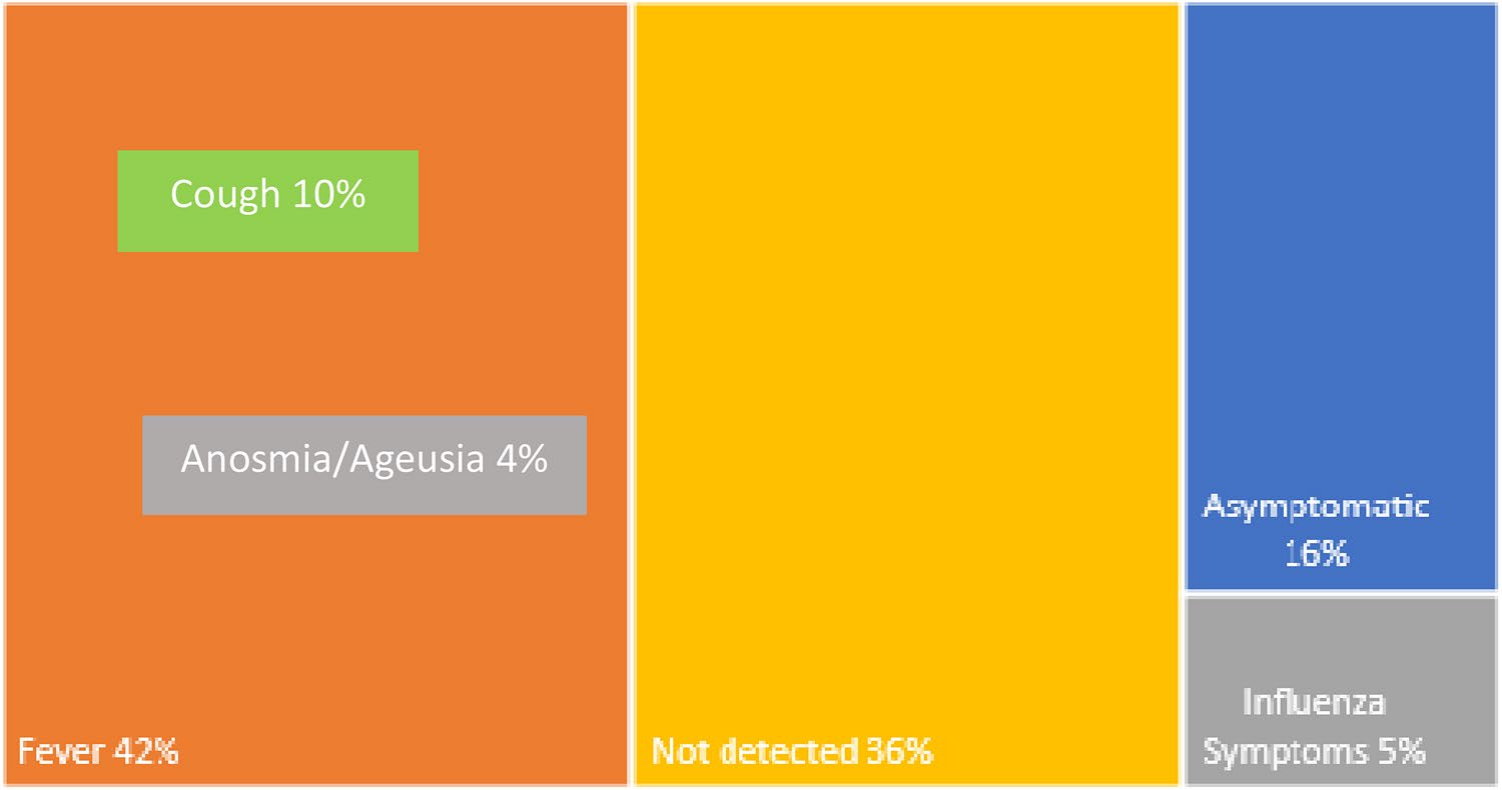
Anamnestic symptoms in IgG positive patients.

Features of IgG positive and IgG negative individuals are represented in Table 2 and Fig. 4.

**Table 2:** Characteristics of the subjects grouped by IgG positivity and IgG negativity, and *p*-values of statistical comparison between groups (bold values are significant).

	IgG negativity	IgG positivity	***p***
Age (yr, mean ± SE)*	56,0 ± 16,5	52,5 ± 12,2	*0.36*
under 30 yr	168	2	*0.018*
30-69 yr	527	17	
over 70 yr	208	0	
Gender (M/F)	445/458	7/12	*0.28*
Relationship			*0.65*
Single	142	1	
Son/daughter	40	1	
Parent	223	2	
Grandfather/grandmather	10	0	
Uncle/Aunt	1	0	
Grandson	2	0	
Husband	127	5	
Wife	100	3	
Not detected	258	7	
Symptoms*			** < *0.001***
None	519	3	
Fever	32	8	
Illness	3	0	
Diarrhea	3	0	
Flu symptoms	43	1	
Sore throat	18	0	
Nasal discharge	9	0	
Anosmia and Ageusia	1	3**	
Cough	18	2**	
Not detected	257	7	
Quarantine*			** < *0.001***
No	639	5	
Yes	7	7	
Not detected	257	7	
Contact with infected subjects*			** < *0.001***
No	637	8	
Yes	9	3	
Not detected	257	8	

**Fever associated.

### Geospatial patterns

**Figure 5:**
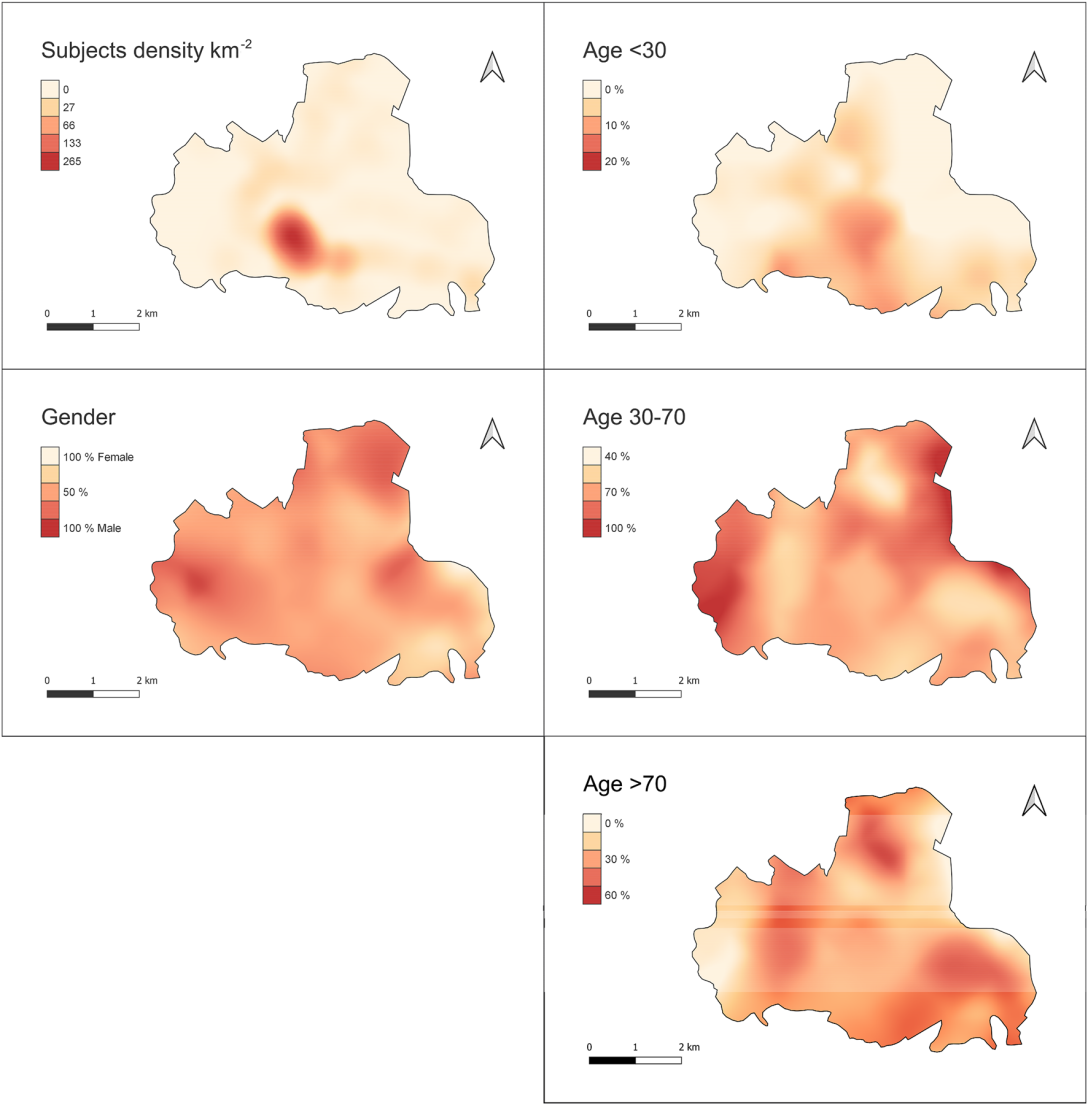
Maps showing the spatial density (individuals km^−2^) of all subjects in the study group and the spatial distribution of gender ratio and percentage of age groups, relative to the tested subjects, *per* km^2^.

As shown in Fig. 5, the significantly higher density of subjects in the town centre dominates the spatial distribution of the study group, with a maximum of about 250 people km^−2^. The density in peripheral areas decreases by a factor of one or more. Females and males are substantially equally represented within the territory, with limited imbalance at the edges (W, N and N-E), where population density is very low. Age groups are unevenly distributed: young subjects reside mainly close to the urban centre; middle-aged subjects are more homogeneously spread (with limited representation at the extreme edges). older subjects reside mostly in the peripheral, rural areas of the Municipality.

Based on the patterns of the bulk data reported above, the following most significant cases were selected for geospatial representation: IgG positive and IgM negative; asymptomatic; fever; flu symptoms; previous quarantine and previous contacts.

**Figure 6:**
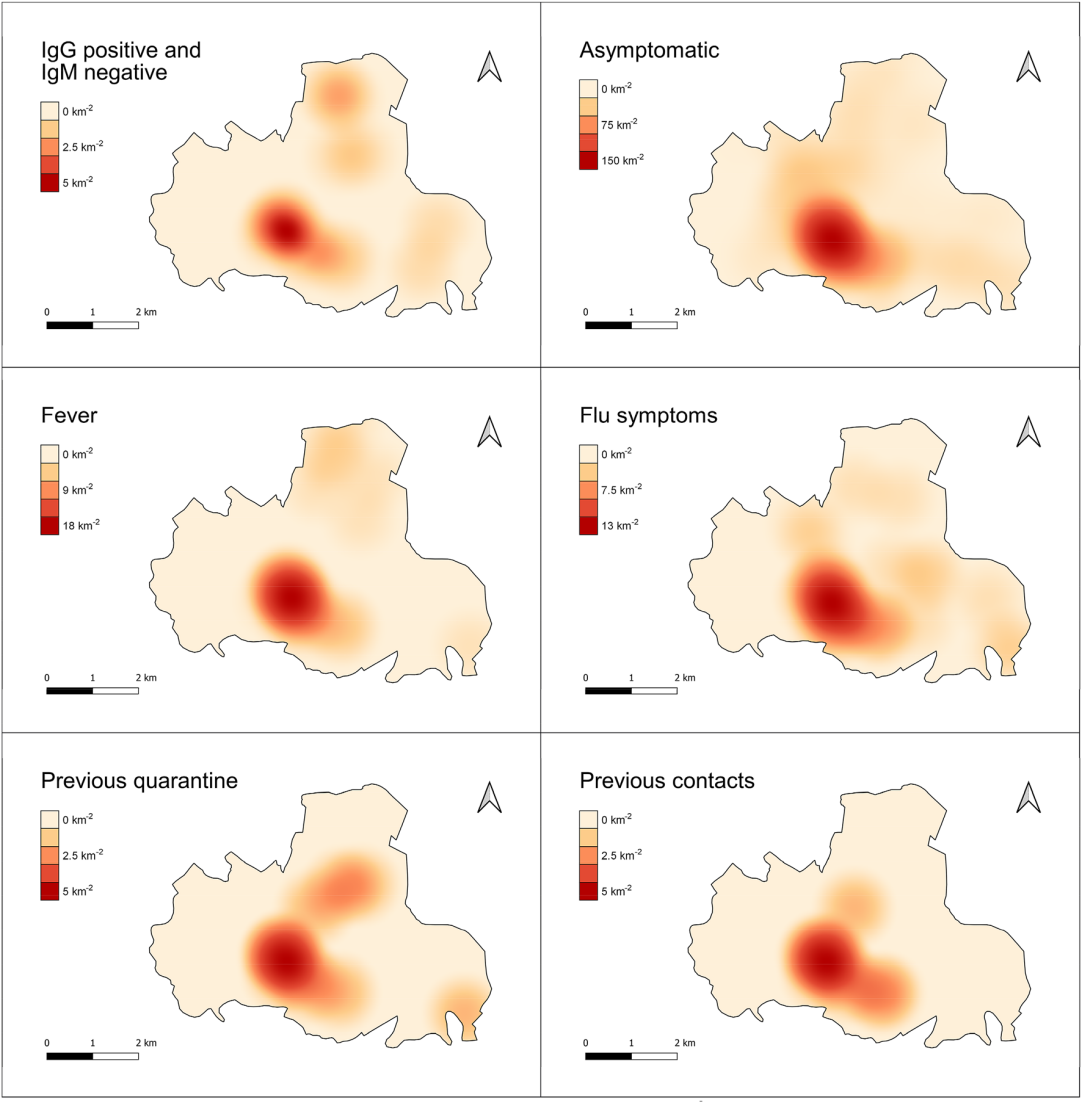
Maps showing the spatial density (individuals km^−2^) of percentage of specific case groups.

As shown in Fig. 6, in absolute terms the density of cases *per* km^2^ reported within all the above categories and the overall density of tested subjects (Fig. 5) are substantially consistent, with most cases concentrating in or near the urban centre, supporting a direct causal relation between such variables. A few peripheral hot spots can be observed, however, for immunised and quarantined subjects only.

**Figure 7:**
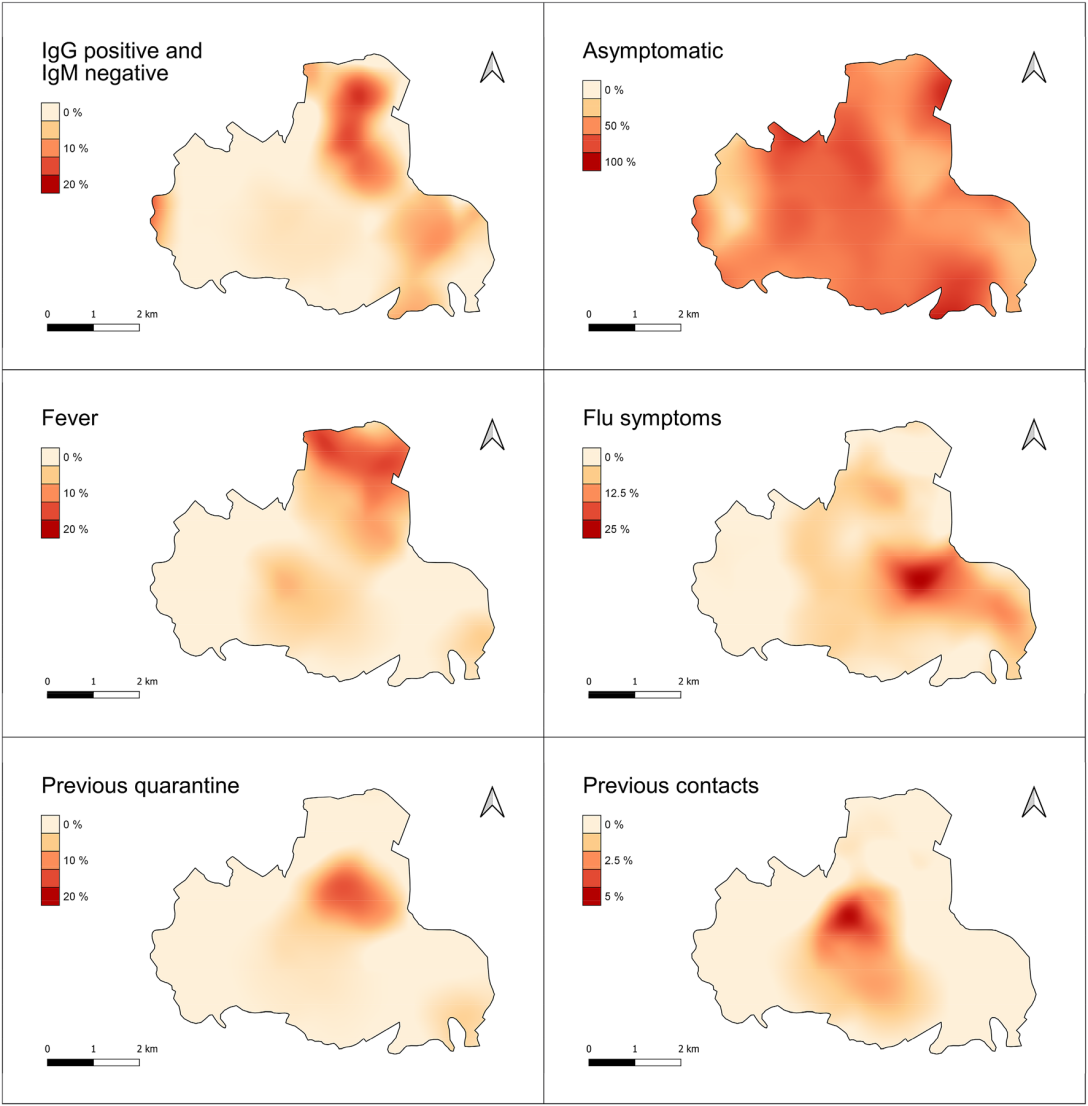
Maps showing the spatial distribution of the percentage of specific case groups relative to the tested subjects, *per* km^2^.

When the density of specific cases is normalised by that of the overall tested subjects (population), further patterns emerge (Fig. 7).

Despite the absolute majority of IgG positive and IgM negative cases (11 out of 19) concentrating in the urban centre, peripheral areas (particularly northern) had a significantly higher relative number of cases compared to the urban centre. This is consistent with the relative proportion of subjects with fever or previously quarantined. The relative percentage of patients reporting flu-like symptoms is also concentrated in a restricted peripheral area nearby (N-E), and so is the relative percentage of patients reporting previous contacts with infected patients from phase 1—in a restricted area between the urban centre and the quarantined hot spot. Of particular interest, all the above case categories exhibited a number of involved subjects for inhabitants higher in peripheral rural zones than in the urban centre. This reflects an older age group (Fig. 5). Asymptomatic tested subjects were homogeneously distributed within the Municipality, showing no spatial correlation with previous groups.

**Figure 8:**
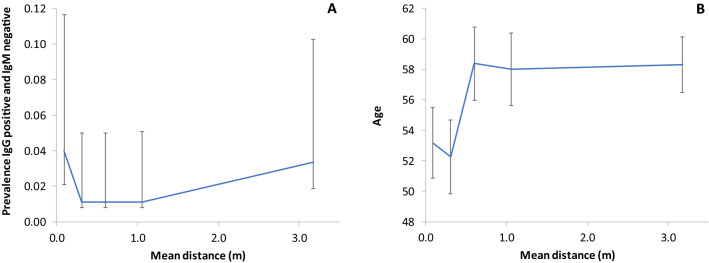
(**a**) Prevalence of IgG positive and IgM negative case as function of their distance (five equi—represented classes) to the nearest phase 1 infected subjects. (**b**) Mean age of subjects within the same distance classes (irrespective of immunity).

Nearest neighbour analysis was performed to assess the degree of spatial association between: immunized subjects, age and phase 1 infected patients, the latter being considered as representative of the overall distribution of (even subclinically) infected population during phase 1, rather than as a direct origin of subsequent transmission. Figure 8a shows that on a qualitative level the prevalence of IgG positive and IgM negative subjects is highest at minimum (< 180 m) or maximum (> 1.3 km) distance from previously infected subjects, and lowest at intermediate distances. The mean age of subjects is notably lower in the closest distance classes (< 440 m), in accordance with the preferential relative concentration of young subjects towards the urban centre (where the average distance between generic subjects reduces), as shown in Fig. 8b.

## Discussion

The present study describes the prevalence of anti-COVID-19 antibodies in a small municipality in the Province of Treviso, in the Veneto Region, describing the epidemiological trend of Coronavirus during the February-May 2020 pandemic. In particular, screening of at least one subject in each family unit allowed us to estimate how the infection may have spread inside the families of symptomatic and asymptomatic subjects. The low presence of subjects with positive IgG and negative IgM (2.1% of the total) can be due either to the study phase subsequent to the pandemic peak in the region and the small area of Veneto. It is demonstrated how this virus has had a different circulation than in other areas of the epidemic and how its distribution in Italy is heterogeneous. Rural areas with low population density, as in Monastier have a lower contagion percentage than the areas with higher population density. The lower morbidity in Northwest China might be attributed to its low population density and geographical distance from the epicenter. A previous study proved that high population densities catalyze the spread of COVID-19^23^.

However, vulnerability in certain urban areas can be driven by several factors such as the presence of overcrowded housing and a relatively above-average prevalence of underlying health conditions. This can account for up to 34% variability^24^.

On the contrary, the serological prevalence observed in Villa Caldari, another small municipality of Abruzzo region was 10.9% which is four times greater than the value (2.5%) reported as preliminary result of a the nation-wide Italian survey on SARS-CoV-2 antibodies. The prevalence observed in the area was also higher than the one estimated for the overall Lombardy region, the most heavily affected region by the pandemic in Italy, which stood at 7.5%. A more similar prevalence was observed in Central Spain and in the area of Madrid (> 10%).

Although transmission dynamics in a small village like Villa Caldari could be very different from what expected in metropolitan areas, where public transportations and other places with a high concentration of people can play a major role in the spread of the infection. In a small village, other aggregative sites, such as bars, pubs, shops, etc., together with the intense social relationships among relatives, may place a crucial role in the transmission of the infection, as demonstrated by the numerous familial clusters identified, as well as the cluster linked to a bar and tobacco shop^25^.

Similarly in our rural area, the subjects who lived with infected people developed immunity in 25% of cases. This indicates how forced coexistence, but respecting hygienic-sanitary and behavioural rules of "isolation within isolation", can reduce infection, when compared to those who had not come to know with certainty that they were living with an infected person. Only 9 subjects with a positive buffer were found in the Municipality of Monastier di Treviso during the Pandemic Phase, with an incidence of 0.2% of the total population. Forced cohabitation in the family environment during the lockdown contributed to the spread of the infection within families, while the isolation of those infected in nursing homes for the elderly protected other residents from the risk of infection (Fig. 1).

Moreover, from the geospatial distribution of the homes of the subjects with positive IgG in the Municipality of Monastier, it is evident that the housing proximity of families affected by Covid is very relevant from an epidemiological point of view (distribution may be defined as a "leopard’s spots"). In fact, subjects with positive IgG lived in or near areas inhabited by positive subjects, as determined by nasopharyngeal swab.

From the epidemiological trend it is possible to deduce from the graph of the prevalence of positive outcomes. These vary as a function of the average distance of the subjects from the nearest positive patient (result positive on NAAT). As prevalence was very low, we cannot identify statistically significant trends. However, in qualitative terms, there is an interesting bimodal trend, with higher prevalence for those who live very close (less than about 200 m) or far (more than about 1.3 km) from a positive buffer zone. In fact, these two factors could represent two different transmission modes: those who live very close to infected people (more likely in urban centres) are more likely to come in contact with the virus due to random proximity effects. Those who live far from infected people (more likely in the suburbs or countryside) have a higher probability to come in contact with the virus due to intentional / relational proximity. Among other things, the calculation of the average age between the distance classes confirms the impression from the maps where the more advanced (more sensitive) age groups tend to be relatively more localised in less densely populated areas. This, combined with the above interpretation, substantially confirms the obvious fact that those who are older usually live in the suburbs (and probably move even less). Also, they encounter transmission more by relational contact than by casual proximity.

In the Spanish cohort there was a higher parental incidence, rather than being linked to a stable family relationship compared to single individuals (0.7%) or grandparents. Living in a context of isolation (even forced) has allowed grandparents to maintain immunological integrity, due to the absence of infection. The subjects who did not remain in quarantine due to the absence of contact with those infected, or did not report contact with the infected, but in forced lockdown, demonstrated a reduced possibility of contagion. By contrast, the subjects in quarantine, having been in contact with an infected person were themselves infected and developed immunity in 50% of cases, even if asymptomatic^25^.

The distribution by age allowed us to understand how the middle age (from 30 to 69 years) was more affected, given the greater frequency of movements of subjects, often for work job reasons, as compared to elderly subjects (over 70), who remained in isolation from the rest of the family. Even in young subjects (under 30) the circulation of the virus was low, probably due to lockdown rules being widely respected. Also, there are fewer younger people in this—mainly rural—municipality. In asymptomatic subjects (522 people, equal to 80% of the population studied with anamnestic collection), only 3 were positive. This demonstrates that if the virus has a low circulation in an area, asymptomatic subjects are less dangerous and lockdown prevents contagion. Their lower mean age, which is a generally associated with better health, could have further reduced risk of severe infection.

Approximately one-third of seropositive participants were asymptomatic in the Spanish-based study similary to this study^25^. A study from Henan Province of China showed that an asymptomatic carrier transmitted COVID-19 virus to her five family members^20^; therefore, the management of asymptomatic patients is critical for preventing outbreaks. Seventeen percent of patients were asymptomatic carriers, all of whom were detected during the period of quarantine or medical observation^23^.

The symptom that was mostly correlated with remote antibody COVID-19 positivity was fever (20% in febrile subjects). Indeed, in 163 symptomatic subjects (17.6% of the total) hyperpyrexia was the most frequent symptom (24.5%). Of these, only 4.9% were IgG positive. On the contrary, subjects with general flu symptoms (27% of the symptomatic ones) antibody positivity to COVID-19 was as low as 0.6%. In the Spanish study by Cito et. al amongst the symptomatic infections, the most frequent symptoms are consistent with COVID-19. In our population, dyspnoea was poorly reported, possibly because outpatients not presenting severe respiratory distress composed it. Anosmia (the loss of the ability to detect one or more smells) should be recognized as an early symptom of COVID-19^25^.

Limitations of this study are related to the difficult diagnostic approach at the beginning of Sars-Cov-2 Pandemia.

## Conclusion

SARS-CoV-2 has caused a global pandemic and is associated with significant morbidity and mortality. Serological tests appear to be unreliable during the first infectious period. However, IgM may play a role in subjects with at least 14 days after the onset of symptoms attributable to Covid-19. The presence of IgG antibodies within a population is useful to understand how widely the virus has circulated in the weeks and months following the epidemic. The heterogenic epidemiological distribution of Sars-Cov2 infection in different rural and metropolitan areas (a "leopard’s spots" distribution) has been demonstrated by different studies. Aside from anamnesis of fever, quarantine and isolation with infected subjects which have been linked to IgG positivity, subjects who live very close to infected people are more likely to come into contact with the virus due to random proximity effects. By contrast, those who live far from infected inhabitants have a greater probability of coming into contact with the virus by intentional / relational proximity, determining the concept of reciprocal spatial distance by categories.
